# Distinct *Cutibacterium acnes* subspecies *defendens* strains classified by multi-omics dissection alleviate inflammatory skin lesions of a rosacea-like mouse model

**DOI:** 10.3389/frmbi.2024.1362408

**Published:** 2024-10-21

**Authors:** Hanseol Kim, Kihyun Lee, Ji Young Lee, Bo Eun Kwon, Hyun Jeong Kim, Hyunkyung Park, Taekyung Kim, Jun-Gu Kwak, Joung Eun Choi, Kwang Hee Hong, Jongsik Chun, Changsik Shin

**Affiliations:** ^1^ Discovery R&D, CJ Bioscience, Suwon-si, Gyeonggi-do, Republic of Korea; ^2^ BIO Digital Platform R&D, CJ Bioscience, Seoul, Republic of Korea; ^3^ Analysis&Quality MFG BIO, CJ CheilJedang, Suwon-si, Gyeonggi-do, Republic of Korea; ^4^ CMC R&D, CJ Bioscience, Suwon-si, Gyeonggi-do, Republic of Korea; ^5^ Culture Ingredient R&D FNT, CJ CheilJedang, Suwon-si, Gyeonggi-do, Republic of Korea; ^6^ CJ Bioscience, Seoul, Republic of Korea

**Keywords:** *Cutibacterium acnes* subsp. *defendens*, genomic heterogeneity, acnecin, ultraviolet, amino acid metabolism

## Abstract

**Introduction:**

*Cutibacterium acnes* (*C. acnes*) resides in various organs such as the skin, prostate, eye, nose, stomach, and intestine, indicating the possibility of extensive crosstalk between this bacterium and the human body. *C. acnes* strains are classified into three subspecies based on phylogenetics and distinguishable phenotypes. Among them, *C. acnes* subsp. *defendens* strains are characterized by anti-inflammatory features, raising expectations for their potential as future microbiome therapeutics. However, the heterogeneity of *C. acnes* subsp. *defendens* and its corresponding immunological functions have not been clearly addressed.

**Methods:**

The genetic diversity of the strains was assessed using single- and multi-locus sequence typing. Their immune-modulatory functions were evaluated in vitro using 2D and 3D assays with immune and epithelial cells. The anti-inflammatory effects were further confirmed *in vivo* using a rosacea-like mouse model. Comparative genomic and transcriptomic analyses were conducted to uncover mechanisms underlying the immunosuppressive activity of the strains.

**Results:**

We demonstrated that the newly isolated *C. acnes* subsp. *defendens* strains, exhibiting phenotypic heterogeneity, are distinctly clustered using single- and multi-locus sequence typing methods. These strains showed strong immune-regulatory functions in immune and epithelial cell-based 2D and 3D *in vitro* assays. Furthermore, their anti-inflammatory role was functionally confirmed *in vivo* using a rosacea-like mouse model, where they alleviated skin lesions characterized by hyperplasia and dermal inflammation. Comparative transcriptomics revealed that these strains may exert their immunosuppressive effects through the enhanced expression of acnecins and transcriptional variation in envelope stress regulators (specifically the two-component systems, CesSR homologs). Additionally, we propose that these *C. acnes* type II strains produce anti-inflammatory metabolites or peptides smaller than 3 kDa, which are associated with elevated pyrimidine and reduced L-arginine biosynthesis.

**Discussion:**

The newly isolated *C. acnes* subsp. *defendens* strains demonstrate significant anti-inflammatory properties both *in vitro* and *in vivo*, suggesting their potential as microbiome-based therapeutics. Their unique genomic and transcriptomic profiles, including the production of small bioactive compounds and specific transcriptomic patterns, underpin their immunosuppressive capabilities. These findings provide a foundation for developing novel treatments for inflammatory skin conditions, such as rosacea.

## 1 Introduction

*Cutibacterium acnes* (*C. acnes*) is a common member of the human skin microbiome and known to have a significant influence on the homeostasis of human skin immune systems (Bruggemann et al., 2021; Shaffer et al., 2022). The interaction of *C. acnes* with the host in skin microbiomes is known to result in both beneficial and detrimental consequences. Beneficial interactions with the human skin include the improvement of the skin barrier, maintenance of skin homeostasis, and regulation of skin immunity (Almoughrabie et al., 2023). On the negative side, *C. acnes* has been shown to contribute to the pathogenesis of acne vulgaris, rosacea, atopic dermatitis, and psoriasis (Contassot and French, 2014). Variability in the influence of *C. acnes* colonization within the skin microbiome might have multiple determinants, such as the presence of environmental stimuli, the state of the immune system, and the diversity among *C. acnes* strains (Conwill et al., 2022). Strains of *C. acnes* display significant phylogenetic and genomic variability (Cobian et al., 2021), and it has been suggested that the features of the skin habitat manifested by this species might be dependent on its subspecies colonizing the skin (Yu et al., 2016).

Based on the sequences of the *recA* and *tly* genes, *C. acnes* can be further narrowed down to six phylogenetic groups (IA_1_, IA_2_, IB, IC, II, and III) (McDowell et al., 2012; Corvec et al., 2019). *C. acnes* strains are classified into three major subspecies, *C. acnes* subsp. *acnes* (also called type I), *C. acnes* subsp. *defendens* (type II), and *C. acnes* subsp. *elongatum* (type III), which possess morphological differences. Alternatively, some researchers have classified the strains into 11,009 ribotypes (RTs) based on 16S ribosomal RNA sequence polymorphism (Fitz-Gibbon et al., 2013). In addition, the multilocus sequence typing (MLST) (McDowell et al., 2013) and single-locus sequence typing (SLST) (Scholz et al., 2014) methods have been utilized to assign clonal complex (CC) or singletons (Dreno et al., 2018). These advanced sequence typing methods have linked a specific phylogenetic position of *C. acnes* to its corresponding habitat. Acne-associated type I (particularly, RT4 and RT5) strains can be readily found in the acne lesion, while *C. acnes* type II strains can be easily identified in the health-associated skin (Yu et al., 2016; Kolar et al., 2019; Cavallo et al., 2022). Type II *C. acnes* are generally recognized as healthy skin-associated strains composed of RT6 and RT2; however, unlike RT6 mainly found in healthy skin, RT2 is evenly distributed in both healthy skin and inflammatory acne vulgaris. For example, ATCC11828, a representative strain of *C. acnes* type II, was isolated from the inflammatory region, and some type II strains having proinflammatory plasmids have been shown to enhance the levels of proinflammatory cytokines (O’Neill et al., 2024). We therefore anticipated that RT2 may contain heterogeneous strains having different genotypes with their corresponding immunological phenotypes.

Patients with skin inflammatory diseases such as acne vulgaris, rosacea, atopic dermatitis, and psoriasis have significantly decreased abundance of *C. acnes* (Rozas et al., 2021). Particularly in the study of rosacea patients, the abundance of *C. acnes* was inversely correlated with disease progression although the phylogenetic subtype of the *C. acnes* populations in these patients has not been addressed in detail (Woo et al., 2020). In the development of rosacea, ultraviolet (UV) light has been known to induce skin inflammation and thought to be the most important environmental factor in inducing the onset of rosacea (Morgado-Carrasco et al., 2021). Interestingly, a recent study showed that both the *C. acnes* subsp. *defendens* XYCM42 strain and its fermented filtrate less than 3 kDa improve skin condition diminishing oxidative stress, erythema, and inflammation (Rhee et al., 2022). Furthermore, fermented products of *C. acnes* were shown to ameliorate UV light-induced skin pigmentation (Kao et al., 2021). We hypothesize that metabolites or peptides less than 3 kDa produced by certain subtypes of *C. acnes* strains have anti-inflammatory properties.

The identification and isolation of *C. acnes* subsp. *defendens* is particularly significant, given its potential therapeutic applications. This subspecies has demonstrated the ability to produce metabolites or peptides smaller than 3 kDa with potent anti-inflammatory properties, making it a promising candidate for future microbiome-based therapies. The novel strains identified in this study not only enhance our understanding of the genetic and phenotypic diversity of *C. acnes* but also provide a valuable resource for developing targeted treatments for inflammatory skin diseases, such as rosacea. By exploring the heterogeneity and immunoregulatory functions of these strains, this research lays the groundwork for leveraging these properties in therapeutic applications, potentially offering new avenues for treating chronic inflammatory conditions.

## 2 Results

### 2.1 Screening of anti-inflammatory *C. acnes* strains from healthy Korean donor skins

A total of 1,735 bacterial strains were obtained from the facial sebum of 73 healthy donors, and then, 166 evenly distributed strains were selected based on 16S rRNA gene sequence identities. We hypothesized that *C. acnes* may secrete small metabolites or peptides, which can penetrate the corneous layer of the skin to regulate immune responses (Bos and Meinardi, 2000). Based on this hypothesis, we cultured the strains in the optimized plant-based media, filtered the supernatants with a 3 kDa size cutoff, and then used the resulting filtrates in various assays to test the immunological effects of the small molecules produced by the strains. To provide a clear summary of the experimental workflow and strain selection process, we have outlined the key stages of strain collection, screening, and application in **Supplementary Table S1**. The table illustrates the progression from the initial collection of 1,735 strains to the final selection of 6 strains, with a focused analysis on 2 of the most promising strains.

**Figure 1:**
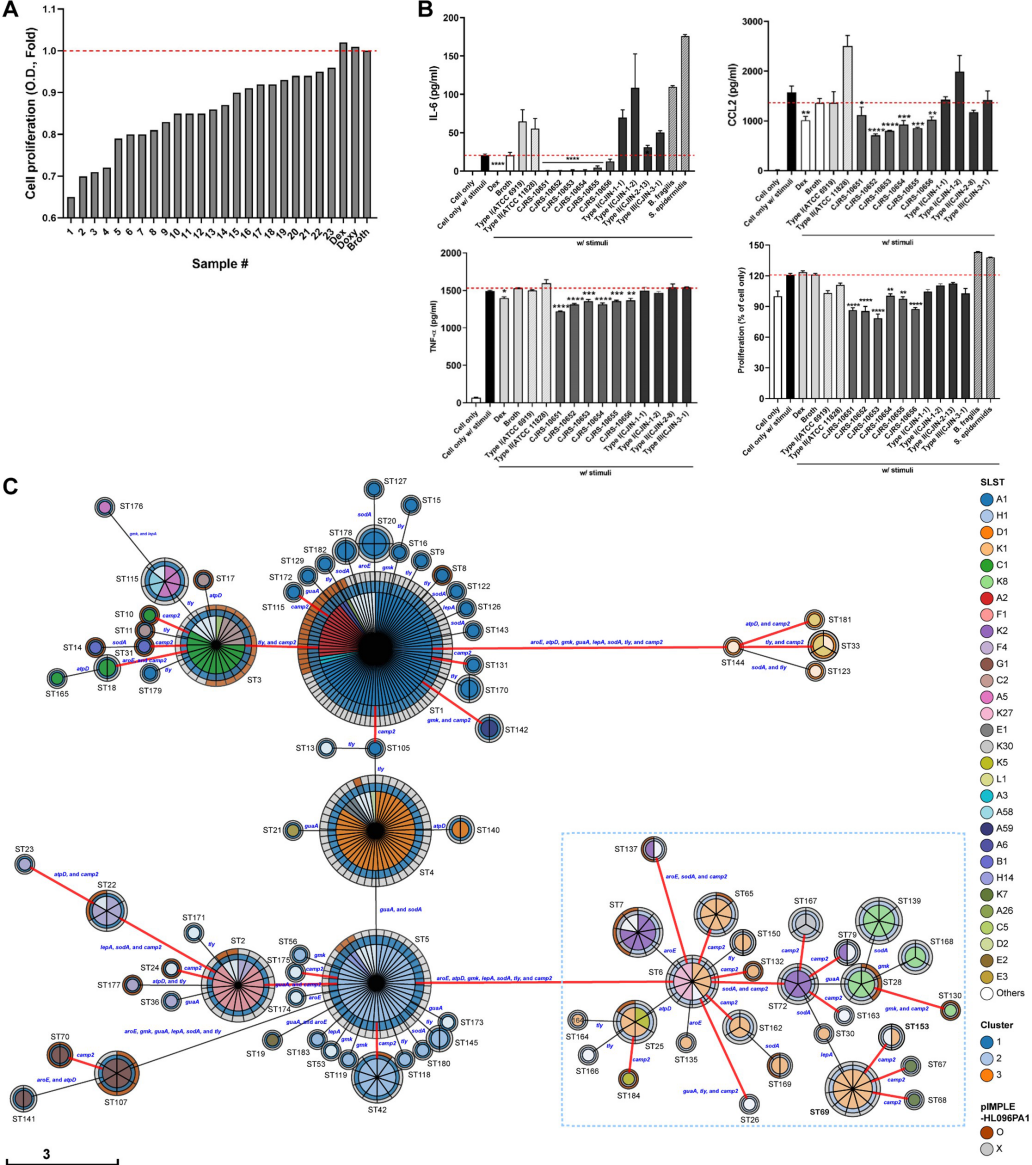
**(A, B)** RAW 264.7 cell line was treated with a 3 kDa filtered supernatant of the indicated bacteria in the presence or absence of stimuli, total supernatant of pathogenic *Cutibacterium acnes*, KCTC3314/ATCC6919. Twenty-four hours after the treatment, cell proliferation of RAW 264.7 was measured via the CCK-8 assay, the cell-cultured supernatant was harvested, and inflammatory cytokines were measured via ELISA. **(A)** A total of 23 *C. acnes* strains were selected, and **(B)** six *C*. *acnes* strains were chosen based on the immune inhibitory levels. Student’s unpaired *t*-test was performed compared to cell only w/stimuli. * <0.05, ** <0.01, *** <0.001, **** <0.0001. **(C)** The genetic relationships among 406 *C. acnes* strains based on genetic typing methods. The minimum spanning tree of 184 sequence types (STs) defined by eight major orthologs was generated using MSTree V2. Each circle represents an ST with its number given next to it, and the size of the circle reflects the number of strains in the ST. The colors in the pie chart indicate the SLST, while the colors in the circle boundaries indicate the three genomic clusters grouped by variable length k-mer of WGS and the existence of the inflammatory plasmid pIMPLE-HL096PA1. The first boundary represents the genomic cluster, and the second boundary indicates the presence or absence of pIMPLE-HL096PA1. The STs of type II strains are denoted by a sky-blue dotted box, and the red lines connect STs divided by the sequence variant of the *camp2* gene. The length of the scale bar is proportional to the number of allelic differences of eight genes.

We measured the effect of our strains on the *in vitro* proliferation and cytokine secretions of macrophages treated with the cultured supernatant from ATCC6919. The ATCC6919 strain belonging to *C. acnes* type IA_1_ and RT1 is the representative type I strain, originally isolated from facial acne (Douglas and Gunter, 1946), and its cultured supernatant has been employed as an inflammatory stimulus in previous studies (Canellas-Santos et al., 2023). We found that 23 *C. acnes* strains significantly decrease the macrophage proliferation compared to that of the broth control (**Figure 1A**). We further narrowed down to six *C. acnes* strains that resulted in the largest reduction in the levels of cytokines (i.e., IL-6, TNF, and CCL2), which was comparable to that of the dexamethasone (Dex)-treated group (**Figure 1B**). Dexamethasone, a corticosteroid, has been utilized as a positive control since it is known as the most potent and well-known immune-suppressive drug for various inflammatory diseases. These six strains displaying potential anti-inflammatory properties were named with “CJRS” as prefix: CJRS-10651, CJRS-10652, CJRS-10653, CJRS-10654, CJRS-10655, and CJRS-10656. The other *C. acnes* strains from the healthy donors, which were screened out, were labeled with the “CJIN” prefix.

We sequenced the whole genomes of the strains screened in this study (*n* = 16) including the six CJRS strains and determined their intraspecies phylogenetic placements among the strain-level diversity of *C. acnes* using publicly available genome sequences (*n* = 390). The analyzed genome sequences were classified into 48 SLSTs and 184 MLSTs. The whole-genome k-mer clustering method clustered all *C. acnes* strains into the three clusters (Xu et al., 2023). The selected CJRS strains all belonged to genomic cluster 2, type II, and RT2 (**Figure 1C**; **Supplementary Table S2**). These strains were ST 69 (MLST) and type K1 (SLST), except CJRS-10653 which was ST 153, K35. According to the estimated whole genomic discriminatory indexes (*D*), type II *C. acnes* strains showed the highest heterogeneity when the combined discriminating methods (MLST and SLST) were applied (*D* = 0.93), which was similarly shown in the recent study (McLaughlin et al., 2022).

The six CJRS strains were more potent in inhibiting inflammatory immune responses and cell proliferation compared to the representative strains of *C. acnes* type I (ATCC6919) and type II (ATCC11828); the CJIN strains belonging to types I, II, and III; and other skin-derived bacterial species including *Bacteroides fragilis* (ATCC25285) and *Staphylococcus epidermidis* (isolated in this study) (**Figure 1B**). It is thus worthy to note that even the same type II (RT2) subspecies possesses heterogeneity in regulating immune responses. For example, the ATCC11828 strain had no immune regulatory effect observed in the indicated markers. Interestingly, the immune inhibitory effect of our CJRS strains was diminished when the 3 kDa filtration step was omitted (i.e., applying whole cultured supernatant to the co-culture). One possible scenario is that the *C. acnes* strains may produce both immune stimulatory and inhibitory factors, and the immune inhibitory factors are small molecules less than 3 kDa (**Supplementary Figure S1A**).

### 2.2 Anti-inflammatory effects on the 3D skin organoid and rosacea-like mouse model

**Figure 2:**
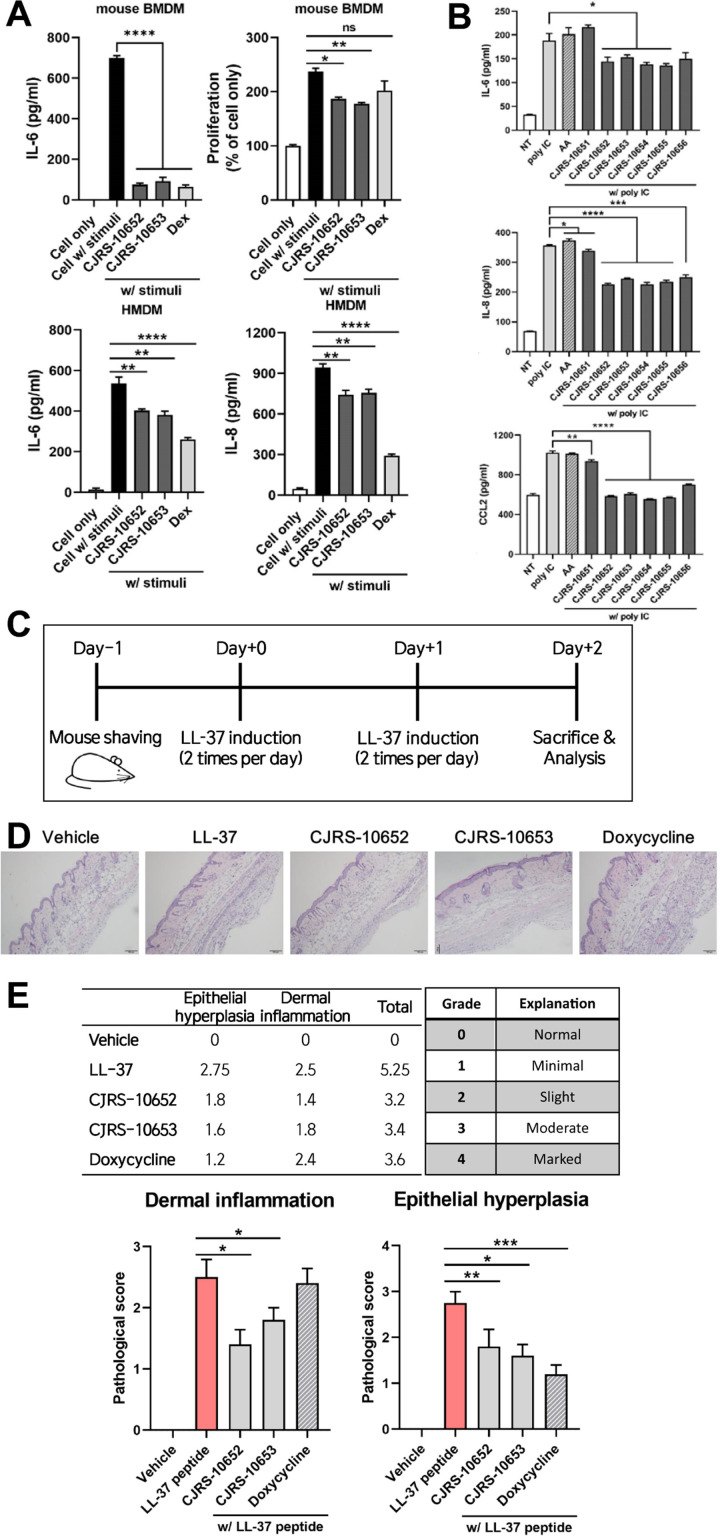
**(A)** BMDM or HMDM was treated with a 3 kDa filtered supernatant of the indicated bacteria in the presence or absence of stimuli, total supernatant of pathogenic *Cutibacterium acnes*, ATCC6919. Twenty-four hours after the treatment, the cell-cultured supernatant was harvested and the indicated inflammatory cytokines were measured via ELISA. **(B)** The 3D organoid was treated with a 5% 3 kDa filtered supernatant of the indicated bacteria in the presence of poly(I:C). Forty-eight hours after the treatment, the cell-cultured supernatant was harvested and inflammatory cytokines were measured via ELISA. AA, ascorbic acid. Pathological scores of rosacea reduced by the treatment of the supernatant of our isolates. **(C)** Experimental scheme of the rosacea-like mouse model was shown. **(D, E)** Representative H&E images **(D)** and pathological scores of epithelial hyperplasia and dermal inflammation **(E)** showed alleviated rosacea skin lesion in the indicated groups. Student’s unpaired *t*-test was performed compared to cell only w/stimuli. * <0.05, ** <0.01, *** <0.001, **** <0.0001. ns, not significant.

We further examined the immunomodulatory effects of the two most immunosuppressive strains, CJRS-10652 and CJRS-10653 (**Figure 1B**), using primary macrophage cells, a 3D skin organoid, and a mouse rosacea-like model. When treated with mouse bone marrow-derived macrophages (BMDMs), the filtrates of the two strains decreased the level of inflammatory cytokine IL-6 and cell proliferation rate dramatically, the efficacy of which was comparable with the Dex treatment (**Figure 2A**). With human monocyte-derived macrophages (HMDMs), we again observed the inhibitory effect of the two strains on the inflammatory cytokines IL-6 and IL-8 although their inhibitory efficacy was lower than that of the Dex treatment (**Figure 2A**).

Next, we utilized a 3D skin organoid to confirm the anti-inflammatory effect of the *C. acnes* strains in a context more resembling the skin–microbe interaction. The organoid model was designed to closely mimic human skin, with a well-controlled and uniform thickness of the matrix. It comprises five distinct skin layers, each well-separated, replicating the structural characteristics of human skin. The organoids were stimulated with poly(I:C) in the presence or absence of the filtrates of CJRS-10652 or CJRS-10653 to examine their anti-inflammatory role in the organoid context. Interestingly, inflammatory cytokines IL-6, IL-8, and CCL2 were significantly decreased when the filtrates were applied, while L-ascorbic acid did not have any effect on them (**Figure 2B**). Ascorbic acid is employed as an antioxidant and a dermal collagen-enhancing agent in various medical and cosmetic applications (Boo, 2022). Owing to its beneficial effects on human skin, ascorbic acid was utilized as a control in our study. We additionally tested the toxicity of the filtrates of CJRS-10652 and CJRS-10653 using the skin epithelial cells. The epithelial cell cytotoxicity was analyzed by applying 5%, 10%, or 20% of the filtrates, and none of the tests showed negative effects on epithelial cell viability (**Supplementary Figure S1B**).

To further assess the immunosuppressive effect of the two strains on *in vivo* skin inflammation, we selected a rosacea-like model since its pathogenesis is closely related to the abundance of *C. acnes*. As previously reported (Li et al., 2020), we confirmed that the LL-37 peptide successfully induces the two major histological phenotypes of epithelial hyperplasia and dermal inflammation for the rosacea-like mouse model. When the filtrates of the two strains were intraperitoneally delivered to the LL-37-treated rosacea mice, both epithelial hyperplasia and dermal inflammation were significantly alleviated, and the level of efficacy was comparable with that of the doxycycline treatment (**Figures 2C–E**), the currently available first-line standard care for rosacea patients.

### 2.3 Genomic features associated with the anti-inflammatory strains

**Figure 3:**
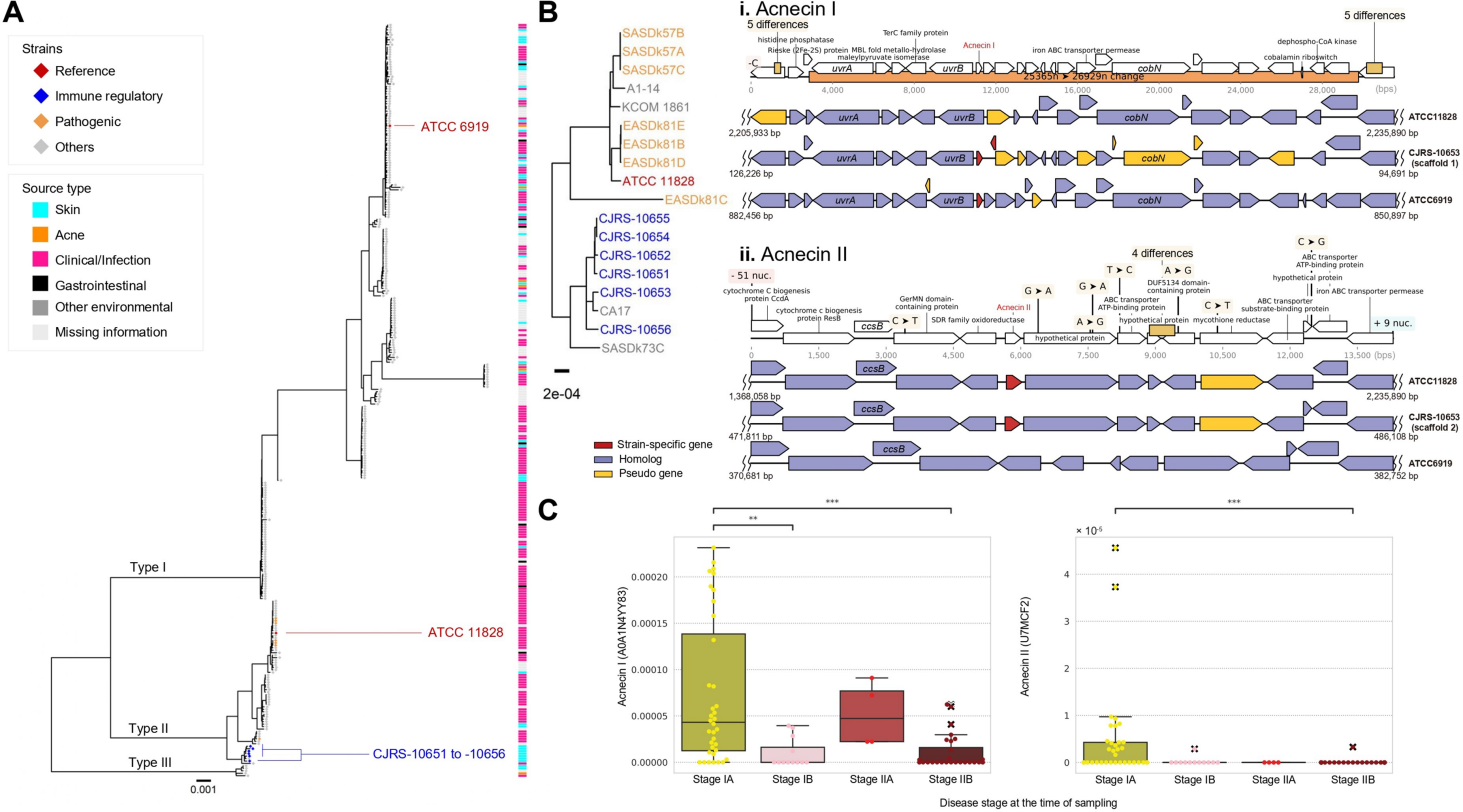
Genomic diversity in the type II *Cutibacterium acnes* strains. **(A)** A maximum likelihood phylogenetic tree was reconstructed by using the core genome alignment and *Cutibacterium namnetense* NTS31307302 as an outgroup. The branches defining the known subspecies were marked by the text label on the branches. **(B)** Comparison of genomes between CJRS-10653, ATCC11828, and ATCC6919 strains identified the common genomic variations. The predicted genes are shown by empty block arrows, and detail variations and gene annotations are indicated by dark brown rectangles and black vertical lines, respectively. The genomic regions related to acnecin I (i) and acnecin II (ii) are depicted. **(C)** The abundance of acnecins I and II in shotgun metagenome data of cutaneous T-cell lymphoma is presented. The reads per kilobase (RPK) values were normalized to relative abundance. The *x*-axis represents the stages of each disease. ** <0.01, *** <0.001.

We conducted a comparative genomic analysis to identify common genomic variations shared among the six anti-inflammatory strains (CJRS strains) that are distinct from those found in the strain ATCC11828, the representative strain of *C. acnes* type II which lacks immunosuppressive phenotypes (**Figure 3A**). A total of 42 and 34 genes were unique characteristics of the six strains and the ATCC11828 strain, respectively (**Supplementary Table S3**). In addition, 17 variations in the translated gene sequences were detected when comparing the six strains to the ATCC11828 strain (**Supplementary Table S3**). The type II RT1 strains have no clustered regularly interspaced short palindromic repeat (CRISPR)–Cas systems, yet the six strains have genetic changes in the *cas3* gene of that region (**Supplementary Table S3**). The six strains have sequence deletions in the 5′ region of CAMP3 and in the 3′ region of CAMP4 (**Supplementary Table S3**). In order to examine whether the six strains possess unique genomic variants when compared to the genomic regions of the previously known type II strains, 62 type II genomes were analyzed, and we found that 12 genomic regions were highly variable, particularly in type II strains (**Supplementary Table S4**). Among the 12 genomic regions, only 4 genes were located on highly variable genomic regions out of 42 unique genes in the six strains and 3 genes were found on them out of 34 genes in the ATCC11828 strain. Moreover, among 17 protein variations, only 2 variations were located on highly variable genomic islands in the six strains. Thus, certain genomic regions of the six strains were found to be specific to a particular focal clade not derived from genomic islands or transferable regions.

We selected high-quality genome assemblies including public genomes (*n* = 106) and examined the distribution of biosynthetic gene clusters (BGCs) among the strains of *C. acnes* (**Supplementary Figure S2A**). We found two types of bacteriocin (acnecin) BGCs which have only 28.79% protein sequence identity to each other (57% query coverage with longer acnecin) within type II *C. acnes* genomes (**Supplementary Figure S2B**). Acnecin I was present in both type I and type II *C. acnes*, while acnecin II was only found in type II strains (**Supplementary Figure S2A**). Interestingly, within type II strains, RT2 has both acnecins I and II, whereas RT6 has only acnecin II, suggesting that RT2 might be the evolutionarily intermediate between type II RT6 and type I (**Supplementary Figure S2B**) (Huang et al., 2021). Based on the phylogenetic features of *C. acnes* subsp. *defendens* particularly with the presence of acnecins I and II, it could be reclassified into three distinct groups as follows (**Supplementary Figure S2B**): RT1 strains with acnecin II, RT2 strains with both acnecins I and II, and RT2 and RT6 strains with only acnecin II (**Figure 3A**). The six immune-suppressive strains have both acnecins I and II with frameshifted genes near acnecin I, whereas the non-immunosuppressive ATCC11828 strain (RT2, ST163, and K9) has only acnecin II (**Figure 3B**).

We suggest that acnecin II is potentially useful as an indicator of type II *C. acnes* as well as healthy skin microbiota. When considering other bacterial species besides *C. acnes*, the acnecin I sequence was frequently observed in the other species, whereas acnecin II is unique to *C. acnes* (**Supplementary Figures S2C, D**). Furthermore, we assumed that the distribution of acnecin II within skin disease patients would vary depending on the onset or severity of the disease, and we tested this hypothesis by analyzing a shotgun metagenomic dataset from a published study on cutaneous T-cell lymphoma (Salava et al., 2020). According to our analysis, acnecin II was found to be more abundant in mycosis fungoides stage IA than in the aggravated stages in which acnecin I is dominant (**Figure 3C**). Although further research is needed, it is speculated that the acnecin II sequence may be utilized as an indicator of healthy skin when analyzing shotgun metagenome data.

### 2.4 Transcriptome signatures of the anti-inflammatory strains

**Figure 4:**
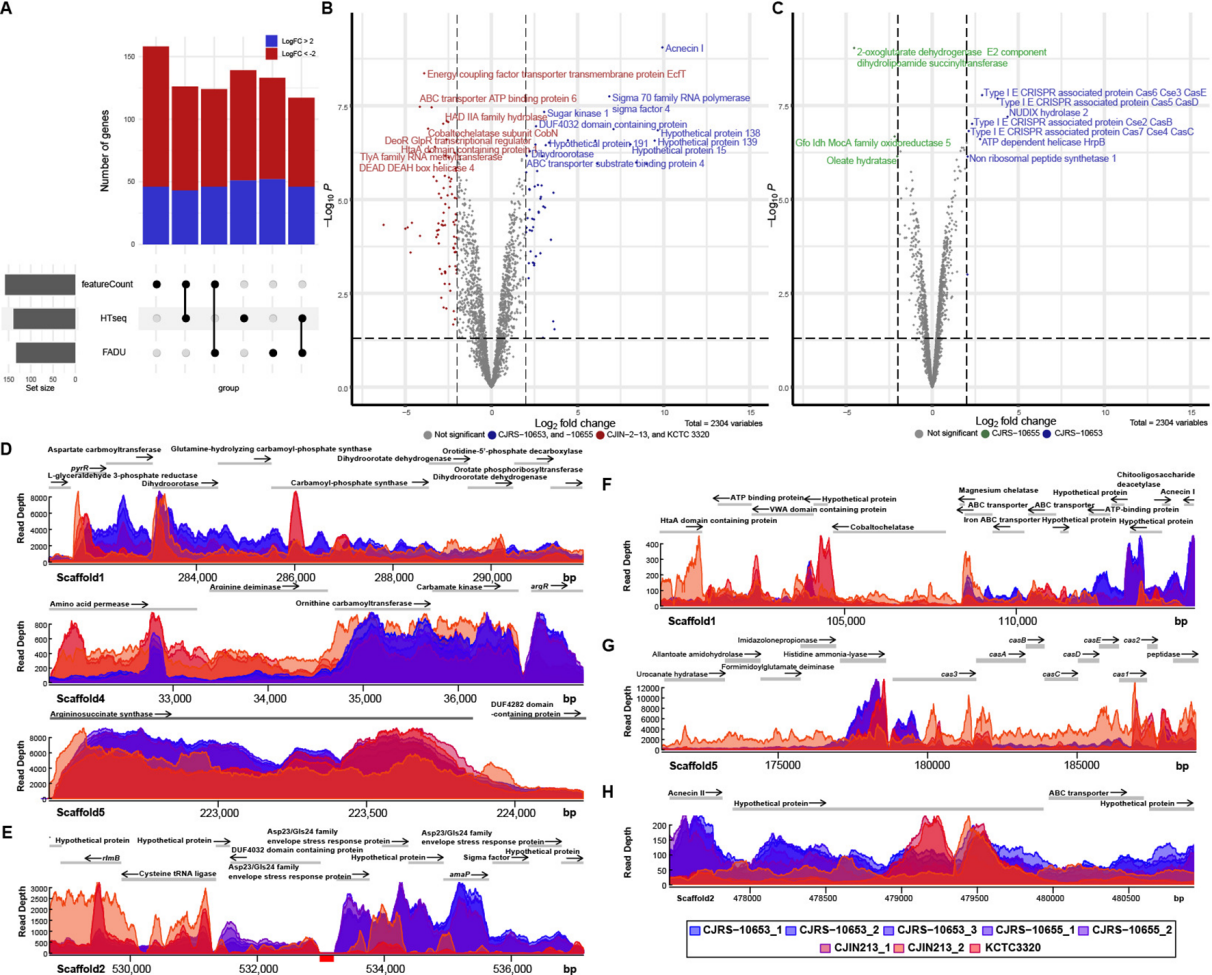
Comparison of transcriptomes among type II RT2 strains. The transcriptomic features of immunosuppressive and non-immunosuppressive strains are colored blue and red, respectively. **(A)** The number of differentially expressed genes (DEGs) was derived from the three methods (featureCounts, HTSeq, and FADU). **(B)** Volcano plot of DEGs between immunosuppressive and non-immunosuppressive strains. The horizontal dotted line indicates the significant threshold (*P* = 0.01), and the two vertical dotted lines represent the two-fold change threshold. **(C)** Volcano plot of DEGs between CJRS-10653 and CJRS-10655. Shown are read depths in the metabolic pathways for L-arginine **(D)**, envelope stress response **(E)**, acnecin I **(F)**, the CRISPR–Cas system **(G)**, and acnecin II **(H)**. **(E)** The genomic coordinate annotated to the cystathionine gamma synthetic gene is represented by a red box at the bottom of the coordinated line.

To seek the unique transcriptional signature of immunosuppressive *C. acnes* strains, we performed comparative transcriptomic analysis of the two phylogenetically distant anti-inflammatory strains, CJRS-10653 and CJRS-10655, and the two non-immunosuppressive strains, CJIN-2-13 and KCTC3320/ATCC11828. Due to the thick cell wall of *C. acnes*, conducting cell lysis for RNA-seq requires certain challenges (Lin et al., 2013). To assess the quality of our sequencing data, we employed FastQC and confirmed that our data quality was not significantly different from that of public RNA-seq data of *C. acnes* (**Supplementary Figure S3A**). The transcriptome profiles of the two anti-inflammatory strains clustered closely on the first two principal components, while the transcriptome profiles of non-immunosuppressive strains were distant from each other (**Supplementary Figure S3B**). The examined biological replicates have a Pearson correlation coefficient of 0.95 or higher (**Supplementary Figure S3C**). We also analyzed published transcriptomic profiles of type I (type IA KCTC3314/ATCC6919 and type IB KPA171202 strains) as well as type II (KCTC3320) strains and found that the transcriptional profiles of our anti-inflammatory strains are closer to those of type II than type I strains (**Supplementary Figure S3C**). When read counts were evaluated through three reference genome alignment-based methods (HTseq, featureCounts, and FADU) (**Supplementary Table S5**), 43 and 70 differentially expressed genes (DEGs) were detected to be enriched and depleted, respectively, in the anti-inflammatory strains (**Figure 4A**, **Supplementary Table S6**). We further identified that the number of DEGs between the anti-inflammatory and non-immunosuppressive groups was higher than that of DEGs between CJRS-10653 and CJRS-10655 (**Figures 4B, C**), visualized in the heatmap and shown in the gene set enrichment (**Supplementary Figures S3D, E**, **Supplementary Table S7**).

Overall, we observed that the *de novo* pyrimidine biosynthesis pathway (**Figure 4D**), the cobalt ion importer (*cbiMNOQ*), the membrane stress response genes (**Figure 4E**), acnecin I (**Figure 4F**), L-serine hydroxymethyltransferase, and the electron transfer flavoprotein (ETF) operon (*etfBA*) were enriched in our six strains, whereas the type IV secretion systems, the HtaA antigen (**Figure 4F**), the *tly* gene, the tripartite biotin transporter (*bioMNY*), and the arginine deiminase pathway were depleted (**Supplementary Table S6**). We also observed enrichment of the *de novo* pyrimidine biosynthesis pathway and the membrane stress response genes as well as depletion of the arginine deiminase pathway, previously known as stationary phase-dependent transcriptions of type I strains (**Supplementary Table S6**) (Brzuszkiewicz et al., 2011), whereas the exponential phase-dependent transcriptions were not changed. The six strains have no L-cysteine gamma lyase (TIIST44_RS12455), while its transcript was detected in the transcriptome of the KCTC3320 strain (**Figure 4E**). Moreover, we observed different transcriptional patterns on L-histidine ammonia-lyase, the signature nuclease (*cas3*) of the CRISPR–Cas systems, and *cas12* genes involved in new spacer acquisition (**Figure 4G**). Interestingly, the expression level of acnecin II is higher in anti-inflammatory strains than that of acnecin II in non-immunosuppressive strains (**Figure 4H**).

**Figure 5:**
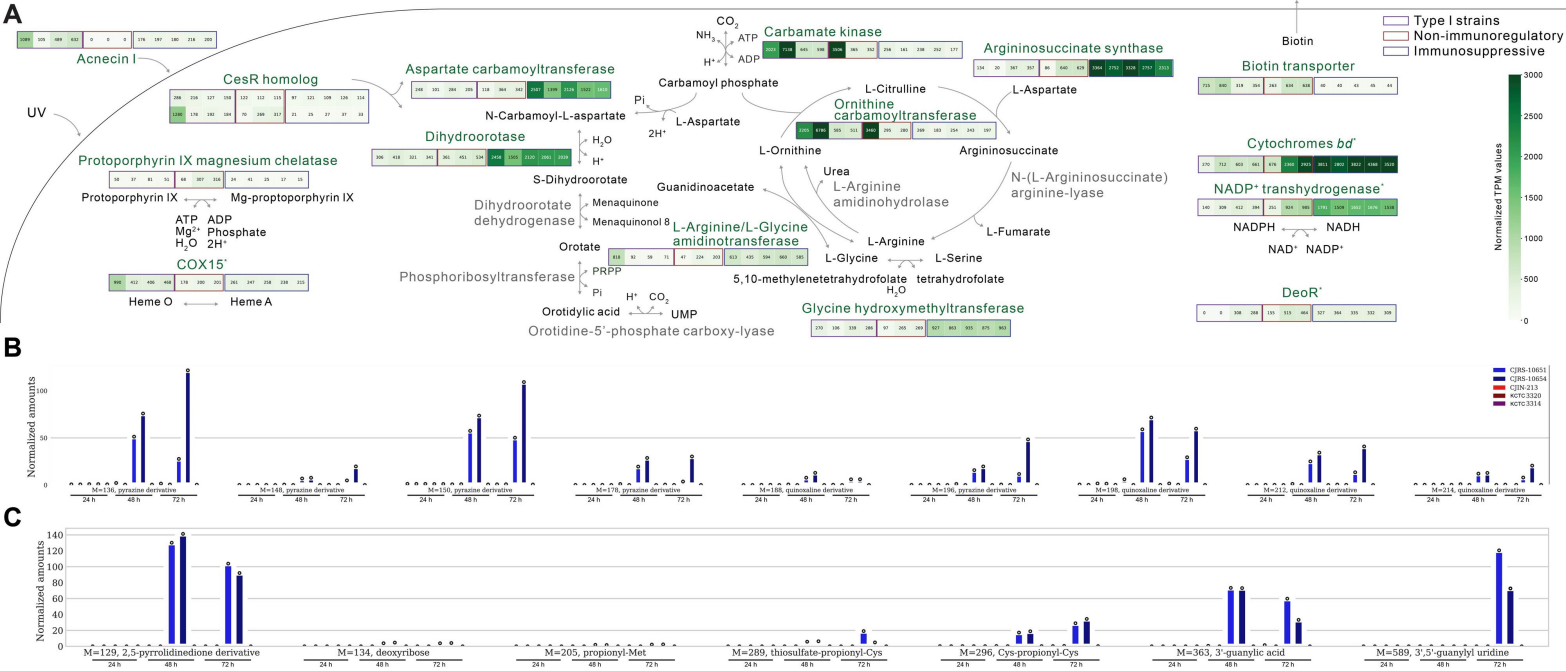
Distinct transcriptomic features of our *Cutibacterium acnes* strains. **(A)** All the indicated transcriptomic differences are significant except for COX15, respiratory-related genes (cytochrome *bd* and NADP^+^ transhydrogenase), and the *deoR* gene. **(B)** The metabolites detected in the culture supernatants of *C*. *acnes* strains in the positive mode of untargeted metabolomics workflow. From left to right on the *x*-axis, the first five points correspond to a 24-h bacterial culture, the middle five points correspond to a 48-h bacterial culture, and the last five points correspond to a 72-h bacterial culture. The results from the supernatants of immunosuppressive type II strains are shown in blue, those of non-immunosuppressive strains are colored red, and those of type I strains are marked purple. **(C)** The metabolites detected in the culture supernatants of *C*. *acnes* strains in the negative mode of untargeted metabolomics workflow.

A previous study has shown that lactococcin 972 influences the bacterial cell membrane by either increasing the expression of envelope stress-related genes or decreasing the expression of pyrimidine biosynthesis-related genes, and it has demonstrated the presence of regulatory proteins CesR regulating envelope stress and pyrimidine biosynthesis-related genes (Marti Nez et al., 2000). While we have identified the presence of the homologous regulatory proteins of CesR in most *C. acnes* genomes (**Supplementary Figure S4A**), our strains had genetic variations in CesR, which might explain the low expression level of this gene in our strains (**Supplementary Figures S4A, B**, **Supplementary Table S5**). The enhanced expression of pyrimidine biosynthesis-related genes in our strains could be driven by the low expression of CesR (**Figure 5A**). Therefore, our strains appear to be capable of overproduction of pyrimidine and acnecin likely due to the lack of an indirect negative feedback (**Figure 5A**). We assumed that expressional patterns of CesSR and its regulons would be closely correlated. To identify regulons of CesR in type I and II *C. acnes*, both positively and negatively correlated genes were unearthed through the chi-squared statistical analysis (**Supplementary Table S8**). Genes with the strongest positive correlation with CesR are the signal recognition particle-docking protein FtsY and alanine/ornithine racemase family PLP-dependent enzyme (**Supplementary Table S8**). FtsY is considered to be essential for the expression of integral membrane proteins, part of the prokaryotic SRP-like machinery (Seluanov and Bibi, 1997). The alanine/ornithine racemase family PLP-dependent enzyme has a 41% protein sequence identity with the ornithine racemase (UniProt accession number: C1FW08) from *Acetanaerobium sticklandii*.

### 2.5 Metabolic states of healthy skin-associated type II *C. acnes* strains

It has been reported that *C. acnes* strains need various amino acids as their essential sources (McCubbin et al., 2020). Through comparative transcriptomic analysis, we observed the different expression profiles in the urea cycle between immunosuppressive and non-immunosuppressive *C. acnes* strains (**Figure 5A**). Interestingly, genes associated with L-aspartate utilization were upregulated in the immunosuppressive strains (**Figure 5A**). L-Aspartate, an amino acid precursor, is utilized for both membrane integrity and pyrimidine biosynthesis, and notably, we found that the selected anti-inflammatory strains have enhanced enzyme expressions related to the L-aspartate pathway (Solopova et al., 2016).

Through comparative genomic analysis, we observed that *argC* gene expression was enhanced in the anti-inflammatory strains although we observed its variations in them (**Supplementary Table S5**). The enzyme encoded by the *argC* gene plays a role in converting L-glutamate to L-ornithine, suggesting its potential influence on L-arginine biosynthesis. In the metabolomic analysis of CJRS-10653 and CJIN-2-13, the anti-inflammatory strain produced more L-ornithine and consumed L-arginine from the growth medium components compared to that of the non-immunosuppressive strain (**Supplementary Figure S5**). The non-immunosuppressive strain secretes L-citrulline (**Supplementary Figure S5**).

In summary, we interestingly observed the importation of L-arginine, L-serine, L-aspartate, L-threonine, L-asparagine, and L-lactate and the secretion of L-ornithine in our anti-inflammatory strains, while the secretion of L-citrulline was shown in the non-immunosuppressive strain (**Supplementary Figure S5**), of which these metabolic pathways need to be further examined to match with their corresponding phenotypes.

## 3 Discussion

Besides MLST and SLST, ribotyping (Fitz-Gibbon et al., 2013), multilocus variable number tandem repeat analysis (MLVA) (Hauck et al., 2015), and bilocus sequencing typing CUTIS–SEQ (Huang et al., 2021) methods have been utilized to address the heterogeneity of *C. acnes*. When ribotyping was developed, 11,009 ribotypes (RT) of *C. acnes* were discovered by analyzing skin metagenomics data (Fitz-Gibbon et al., 2013). However, the most abundant RTs (RT1, RT2, and RT3) were evenly distributed between acne patients and normal individuals (Fitz-Gibbon et al., 2013). With the MLVA method, the variable number of tandem repeat (VNTR) polymorphism was identified in *C. acnes*, but due to its low typeability, it is yet unclear to link between MLVA types and clinical outcomes (Hauck et al., 2015). In this study, we have applied the combination of three algorithms (MLST, SLST, and k-mer-based clustering) to resolve the genetic heterogeneity of *C. acnes* strains and to associate those genetic types with the heterogeneity of anti-inflammatory phenotypes observed among the *C. acnes* strains.

We observed variations in the MLST sequence types of type II strains influenced by the *camp2* potential virulence factor gene (**Figure 1**). In a previous study, *camp5* and *tly* had similar levels of sequence polymorphism, which can result in low antigenic diversification that may lead to mucosal membrane infection (Lomholt and Kilian, 2010). In our transcriptomic analysis, the expression of putative hemolysin *tly* is depleted and that of the surface-exposed putative virulence factor *camp5* is enriched only in the anti-inflammatory strains. The observed expressional differences of *camp5* and *tly* suggest that pathogenic diversification may be dependent on *C. acnes* subspecies.

Among the three major subspecies, *C. acnes* subsp. *defendens* (type II) is known to be the ancestor of *C. acnes* based on the genomic existence of CRISPR–Cas systems and has the most diverse genomic features (Scholz et al., 2016). There have been reports of genomic diversity in the CRISPR–Cas region among type II strains (Bruggemann et al., 2012). The CRISPR–Cas system allows strains to memorize information from genomic parasites, and it has been observed that CRISPR–Cas spacers of *C. acnes* primarily contain sequences from type I strains (Bruggemann et al., 2012). Interestingly, based on our transcriptomic results, the isolated immunosuppressive strains have lower expression levels of the CRISPR–Cas region than the non-immunosuppressive strains although they both belong to type II subspecies (**Figure 4G**).

Since type II strains with the RT1 allele still have acnecin II, analyzing the presence or absence of acnecin II may be more useful to accurately identify type II strains rather than sequencing the 16S rRNA allele (**Supplementary Figure S2B**). While the 16S rRNA gene sequence is approximately 1500 base pairs long, the acnecin II sequence is significantly shorter at 348 base pairs. This makes it a potential marker for shotgun metagenomic, PCR, and/or qPCR markers in detecting *C. acnes* type II strains. Our results show that the presence of acnecin II is specific to type II strains. While type II strains are being considered as a microbial biomarker of healthy skin, it is still difficult to differentiate them from type I strains using the current 16S rRNA gene microbiome method. The reason is that although the 854^th^ nucleotide of the 16S rRNA gene sequence distinguishes RT1 from RT2, the commonly used variable V3–V4 regions (from 337^th^ to 805^th^) cannot cover this position (Fitz-Gibbon et al., 2013). The method we have presented is also important for more precisely quantifying microbiome friendliness (van Belkum et al., 2023).

The immunosuppressive substances of *C. acnes* have not been clearly addressed. It has been recently shown that the type II strain-derived metabolites/peptides less than 3 kDa have considerable antioxidant activity, different from the secreted antioxidant RoxP (16 kDa) by type I strains (Rhee et al., 2022). Since the immunosuppressive effect was observed from the 3 kDa filtrates used in this study, we could infer that the small peptides or metabolites are effector molecules. Through WGS-based comparative analysis, acnecin I was particularly enriched in our six strains among the type II strains. Acnecin I was reported in 1978 as an antimicrobial peptide, but its function has not been clearly understood (Fujimura and Nakamura, 1978). Acnecins I and II are the homologs of lactococcin 972 (class IIb or IIc bacteriocin-like substance) (**Supplementary Figures S2C, D**). Class IIb bacteriocin of *Lactiplantibacillus plantarum* has been reported to have an anti-inflammatory role, reducing proinflammatory cytokines induced by damage in the epithelial barrier integrity (Heeney et al., 2019). Although lactococcin 972 is approximately 12 kDa, it is expected to be cleaved and can pass through the membrane by its cell-penetrating potential. When the filtrates were experimentally tested using MALDI-TOF, a specific peptide peak was observed (data not shown), yet further research is required. On the other hand, the sets of acnecin may be responsible for driving the distinct morphology of *C. acnes* subspecies by a differentially regulating septum (Marti Nez et al., 2000). When we conducted an identification of metabolites using the untargeted method, we observed several nitrogen-containing heterocyclic compounds like pyrazine, pyrimidine, and pyrrolidine derivatives in the supernatants of our strains (**Figures 5B, C**). Interestingly, heterocyclic compounds containing nitrogen have been reported to be promising anti-inflammation agents (Myriagkou et al., 2023; Shin et al., 2023). Unlike type I strains adapted to an oxygen-rich environment, type II strains may not transcriptionally respond to oxygen due to its nature, inhabiting deeper skin (Andersson et al., 2019). Interestingly, the enhanced dihydroorotate dehydrogenase and L-serine hydroxymethyltransferase in our selected strains are known for the *de novo* synthesis of uridine in an oxygen-dependent manner (Peeters et al., 2021) and thymidine synthesis by converting tetrahydrofolate to 5,10-methylenetetrahydrofolate (Anderson et al., 2012), respectively, to potentially provide overproduction of exogenous pyrimidine. Furthermore, anti-inflammatory strains with low CRISPR–Cas region expression have high *hutH* expression. HutH converts L-histidine to *trans*-urocanate (Consevage and Phillips, 1990). *Trans*-urocanate transforms into *cis*-urocanate upon UV exposure, and *cis*-urocanate exhibits anti-inflammatory properties in human inflammatory disease (Kammeyer et al., 2012).

Exogenous pyrimidine has been shown to have several benefits in absorbing UV light and has immunosuppressive effects (Cavalieri et al., 1948; Sprenger et al., 2021; Cicko et al., 2015). Type I *C. acnes* strains are known for producing porphyrins that react with UV light causing inflammation, whereas type II strains express a repressor called *deoR*, which inhibits porphyrin biosynthesis (Mayslich et al., 2021) (**Figure 5A**). Moreover, L-serine hydroxymethyltransferase upregulates the production of vitamin B_6_, which may potentially work as a scavenger of reactive oxygen species induced by UV-mediated damage (Krautkramer et al., 2021; Calori et al., 2021). Sunscreen has been used to prevent UV radiation-induced erythema and edema (Smith et al., 2023), but it does not have a role in immune regulation like our filtrates. We anticipate that our type II *C. acnes* strains secrete potential substances that could alleviate damages caused by UV light in the deep tissues of the skin.

These novel strains possess anti-inflammatory functional features that were observed *in vitro* with two- and three-dimensional assays with significantly decreased levels of several inflammatory cytokines, the efficacy of which was further confirmed with the rosacea-like animal model experiment. Although we have here shown the potential of the particular type II RT2 strains in regulating immune responses, key factors reserved in the strains and their relevant mechanistic action need to be further investigated. The severity of rosacea patients is reported to be reversely correlated with the abundance of *C. acnes* (Woo et al., 2020). Patients face limited treatment options, with current medications showing less than 50% remission rates and a recurrence rate exceeding 50% once treatment is discontinued (Valentin et al., 2009; Webster et al., 2017). The main environmental stress factor in rosacea patients is UV radiation (Salzer et al., 2014). UV radiation primarily induces proinflammation and proangiogenic effects, which can also stimulate the expression of LL-37, an antimicrobial peptide produced by human normal keratinocytes (Kim et al., 2005). While LL-37 is intended to be expressed to prevent the invasion of external bacteria during inflammation, its excessive expression could aggravate the disease state (Salzer et al., 2014). As previously reported (Yoon et al., 2021), the LL-37 peptide successfully induced rosacea-like skin inflammation. Interestingly, the filtrates of our strains successfully diminished LL-37-mediated inflammation, the efficacy of which was comparable to the currently available standard of care doxycycline, demonstrating the potential of developing microbiome-based drugs as additional therapeutic options to reduce the reoccurrence rate of rosacea. Thus, uncovering the distinct strains of *C. acnes* responsible for alleviating the symptoms and lesions of rosacea in the clinic would be a further invaluable study.

## 4 Materials and methods

### 4.1 Bacteria isolation, culture, and supernatant preparation

Skin bacteria were obtained from the nose of 73 healthy donors through skin surface biopsy with D-Squame disc (D100, Clinical and Derm Inc., USA) and cyanoacrylate from CRA Korea, with approval number IRB20-030502. Briefly, D-Squame disc, an adhesive tape with a drop of cyanoacrylate, was utilized to uniformly sample facial skin sebum. The obtained samples were cultured in reinforced clostridial medium (RCM) for 2 days at 37°C, and then 100-fold dilution of the cultured medium was streaked onto a brain heart infusion (BHI) agar plate followed by 2 days of culture at 37°C. The observed microbial colonies were identified by 16S rRNA gene sequencing, cultured in RCM, stocked with 10% glycerol, and then utilized for this study. The isolated strains used in this study were obtained from the Cell Bank of CJ Biosciences, Inc. Type strains of *C. acnes* (KCTC3314/ATCC6919 and KCTC3320/ATCC11828) were purchased from the South Korean Collection for Type Cultures (KCTC, South Korea), and the *B. fragilis* type strain ATCC25285 was purchased from the American Type Culture Collection (ATCC, USA). *Bacteroides fragilis* was grown in tryptic soy broth, and *S. epidermidis* obtained from the Cell Bank of CJ Biosciences was grown in reinforced clostridial broth. All *C. acnes* strains were grown on a reinforced clostridial agar plate (BD, USA) for 4 days and cultured under anaerobic conditions with GasPak (Mitsubishi Gas Chemical, Japan) at 37°C. A single colony was picked up with a sterilized tip and inoculated into 5 mL of reinforced clostridial broth (BD, USA) for 24 h, and the cultured broth was inoculated into a plant-based medium (1%, v/v) and then incubated for 48 h. The composition of the plant-based media is 5 g/L of glucose, 25 g/L of peptone from vegetable, 5 g/L of sodium chloride, 3 g/L of sodium acetate, and 0.5 g/L of cysteine HCl. The bacterial cultured supernatants were prepared as follows: the 48-h cultured broth was centrifuged at 4,000 rpm for 20 min at 4°C. The supernatants were harvested and adjusted to pH 7.0 with 1 N of sodium hydroxide. The pH-adjusted supernatants were filtered with a 0.2-μm disposable membrane filter and then cut off with a 3 kDa size filter (Amicon, USA) by centrifugation. The 3 kDa cutoff filtrates were stored at −20°C and used for the experiment.

### 4.2 Mammalian cell culture

RAW 264.7 cell line was cultured in Dulbecco’s modified Eagle medium (DMEM, Gibco, MA, USA) supplemented with 10% fetal bovine serum (FBS, Gibco, MA, USA) and 1% antibiotic–antimycotic (anti–anti, Gibco, MA, USA) in a 37°C incubator containing 5% CO_2_. For the experiments, RAW 264.7 cells were seeded at 5 × 10^4^ cells/well in 96-well flat bottom plates. Bone marrow cells were isolated from tibias and femurs and cultured as previously reported (Ying et al., 2013). Briefly, bone marrow cells were cultured in RPMI (Gibco, MA, USA) supplemented with 10% FBS, 1% anti–anti, and 10 ng/mL of mouse macrophage-colony-stimulating factor (M-CSF, PeproTech, NJ, USA) for 7 days in a 37°C incubator containing 5% CO_2_. On day 4, the cultured media was changed with fresh RPMI (Gibco, MA, USA) with 10 ng/mL of mouse M-CSF (PeproTech). On day 7, floating cells were removed with phosphate-buffered saline (PBS) and only the attached macrophages were gently harvested with a scraper and then used for the experiments with 5 × 10^4^ cells/well in 96-well flat bottom plates. Human monocytes were purchased from the ATCC and cultured as previously reported (Jaguin et al., 2013). Briefly, monocytes were cultured in RPMI supplemented with 10% FBS, 1% anti–anti, and 50 ng/mL of human M-CSF (PeproTech, NJ, USA) for 7 days in a 37°C incubator containing 5% CO_2_. On day 4, the cultured media was changed with fresh RPMI with 50 ng/mL of human M-CSF. For the experiments, HMDMs were seeded at 5 × 10^4^ cells/well in 96-well flat bottom plates. The HaCaT cell line was maintained in DMEM supplemented with 10% FBS and 1% anti–anti in a 37°C incubator containing 5% CO_2_. For the experiments, HaCaT cells were seeded at 2 × 10^4^ cells/well in 96-well flat bottom plates and at 1 × 10^5^ cells/well in 24-well flat bottom plates.

### 4.3 *In vitro* immune assay

Immune cells were co-cultured with a 10% 3 kDa filtered or non-filtered supernatant of the bacteria in the presence or absence of 2.5% total supernatant of the cultured pathogenic *C. acnes* KCTC3314 strain as stimuli for 24 h. After the co-culture, the rate of cell proliferation was measured by using the CCK-8 assay, and inflammatory cytokines in the cultured supernatant of the co-culture were measured by ELISA. Uncoated mouse or human ELISA kits (Invitrogen, USA) were utilized to measure the level of the indicated cytokines following the manufacturer’s instructions. Cell viability or proliferation was determined by the Cell Counting Kit-8 (Dojindo, USA).

### 4.4 Whole-genome sequencing and analysis

The whole-genome sequences (WGSs) of 16 strains (isolated in this study) were constructed using the Illumina sequencing platform (NovaSeq 6000). For genomic DNA extraction, we used the Wizard genomic DNA purification kit of Promega, WI, USA. For Illumina library preparation and sequencing, the libraries were prepared for 151-base pair (bp) paired-end sequencing using the TruSeq Nano DNA sample prep kit. Sequencing was progressed as paired-end (2 × 151 bps). The average 10 Gbps Illumina raw data were produced. We used quality trimming and adapter clipping with Illumina short-read sequences by the Trimmomatic tool (Bolger et al., 2014). For the construction of assembled genomes, we conducted a short-read-based assembly by SPAdes (Bankevich et al., 2012). Gene annotation files were constructed by the prokaryotic genome annotation pipeline (PGAP) (Tatusova et al., 2016). The sequence types of those WGSs were identified using the expanded MLST *C. acnes* scheme and database (McDowell et al., 2012). Only single isolate genomes were analyzed excluding metagenome-assembled genomes for the true combination of multilocus alleles. The minimum spanning tree (MSTree V2) was constructed and visualized by the GrapeTree tool (Zhou et al., 2018). The phylogenetic tree was constructed using *C. acnes* strains and inferred using IQ-Tree 1.6.12 with the best-fit model (Nguyen et al., 2015). To obtain clusters of genomes, we calculated the MinHash sketch distance and applied the graph-based linear space clustering method based on the minimum spanning tree (Xu et al., 2023). The distance threshold for cluster identification was set at 0.01 during the clustering process. The highly genomic variable regions were predicted by panRGP with default options (Bazin et al., 2020). Biosynthetic gene clusters (BGCs) of *C. acnes* were explored by using BLASTP search with sequences obtained from previous literature (Claesen et al., 2020). To annotate protein sequences, we used Prokka annotation with default option (Seemann, 2014). For the annotation of the acnecin II locus, we conducted a homology search with a protein sequence of acnecin I (Claesen et al., 2020). When BLASTP search was exploited, the potential homologs were identified applying an *E*-value <1 × 10^−2^, a filter for percent identity with ≥40%, and query and subject coverage with ≥80%, whereas BLASTN searches apply specific thresholds (an *E*-value <1 × 10^−2^, a filter for percent identity with ≥70%, query and subject coverage with ≥80%).

### 4.5 Total RNA isolation, sequencing, and transcriptomic analysis

Total RNAs were extracted from the bacterial cell pellets using a bead beater and the Qiagen RNeasy mini kit. The ribosomal RNAs were removed from the extracted total RNA using the Ribo-Zero H/M/R kit. The cDNA libraries were constructed using the TruSeq Standard Total RNA Sample Prep Kit. The amount of cDNA was measured using the KAPA library quantification kit, and the quality was measured using the Agilent 2100 BioAnalyzer. Sequencing of the cDNA library was performed as paired-end (2 × 151 bps) using the Illumina NovaSeq 6000 platform. The quality of RNA sequencing raw data was checked using the FastQC command (Leggett et al., 2013). The adapter sequences were removed using Cutadapt (Kechin et al., 2017). The sequence information of the adapters is as follows: the adapter of the forward read is “AGATCGGAAGAGCACACGTCTGAACTCCAGTCAC” and that of the reverse read is “AGATCGGAAGAGCGTCGTGTAGGGAAAGAGTGTAGATCTCGGTGGTCGCCGTATCATT.” The Illumina short-read sequences were cleaned by the Trimmomatic tool (Bolger et al., 2014). Deduplication was performed using markduplicates of the Picard tool (McKenna et al., 2010). The sequence reads were mapped onto the reference genome sequence by Bowtie2 with a “very sensitive” option (Langmead and Salzberg, 2012). The 5′–3′ biases of the samples were monitored by Qualimap (Garcia-Alcalde et al., 2012). The bed graph files were constructed by bedtools (Quinlan and Hall, 2010). The transcriptomic profiles of the KCTC3320, KCTC3314, and KPA171202 strains were obtained from the publicly available NCBI SRA database (data accession numbers: SRR20999605, SRR20999606, SRR850806, and SRR850808) (Brzuszkiewicz et al., 2011; Lin et al., 2013; Kim et al., 2023). The weakly correlated samples were excluded. The CJRS-10653 genome sequence was used as reference. The number of mapped reads was measured by three reference alignment-based count algorithms: HTSeq (Anders et al., 2015), featureCounts (Liao et al., 2014), and FADU (Chung et al., 2021). The number of mapped reads was normalized to the trimmed mean of M-values (TMM), and DEGs were identified using the edgeR R library method (Robinson et al., 2010). The genes with expressions that were significantly either increased or decreased in the immune regulatory strains were used for further gene set enrichment analysis using FUNAGE-Pro (de Jong et al., 2022).

### 4.6 Metagenomic analysis

Shotgun metagenomics sequencing reads were downloaded from the NCBI SRA database (project name: PRJEB36953). The adapter trimming was exploited using Cutadapt v2.10 (Martin, 2011). The sequencing reads mapped to the host genome were filtered using Bowtie2 v2.4.5 with GRCh38.p13 genome sequence (Langmead and Salzberg, 2012). The taxonomic abundances were calculated by MetaPhlAn v3 and ChocoPhlAn v20191b (Blanco-Miguez et al., 2023). The gene abundances were evaluated by HUMAnN v3.0 and UniRef90 v20191b (Franzosa et al., 2018). Homologs of acnecins I and II were identified using BLASTP search applying an *E*-value <1 × 10^−2^, a filter for percent identity with ≥40%, and query and subject coverage with ≥80%.

### 4.7 Metabolome quantification

Metabolite quantification was carried out using Xevo G2-XS quadrupole time-of-flight mass spectrometry (Waters, MA, USA). Bacterial filtrate (1 μL) was injected onto a Waters ACQUITY HSS T3 (C18, 150 × 2.1 mm, 1.8 μm) column and resolved with a binary gradient, where mobile phase A was 0.1% formic acid in distilled water and mobile phase B was acetonitrile. The gradient began at 98% A, held for 4 min and then ramped to 70% B until 9.0 min, held for 5 min, and returned to 98% A at 14.1 min until 20.0 min for column re-equilibration. The flow rate was 0.3 mL/min, and the column temperature was 40°C. The relative amounts of bacterial metabolites were calculated using a mass spectrometer operated in positive and negative ion modes.

### 4.8 The rosacea-like mouse model and treatment

The LL-37 peptide was utilized to establish a rosacea-like mouse model in BALB/c background (Yoon et al., 2021), and the filtrates of two strains were prepared to examine their *in vivo* efficacy. BALB/c (6-week-old) mice were purchased from Koatech (South Korea). LL-37 was synthesized by Peptron (South Korea) and identified by high-performance liquid chromatography (HPLC), and its purity was over 95%. To conduct a rosacea-like mouse model, mice back skins were shaved 24 h before LL-37 injection, and the mice were intradermally (i.d.) injected with 50 μL of LL-37 (320 μM) twice a day for 2 days with 12-h intervals. Then, the mice were intraperitoneally (i.p.) administered with 150 μL of bacterial filtrates or 1 mg/kg of doxycycline once a day for 2 days. The skin lesions of the mice were evaluated by histology analysis. Isolated skin dermal tissues were fixed with 4% formaldehyde solution overnight, embedded into paraffin and sectioned at 5 μm, and then stained with hematoxylin and eosin (H&E). H&E-stained slides were scored by pathologists blinded to the *in vivo* results. All animal experiments were approved by the Animal Ethics Committee of Woojung Bio (South Korea), IACUC2004-015.

### 4.9 Organoid immune assay

A skin organoid consisting of human fibroblasts and keratinocytes (Lonza, Switzerland) was prepared by 3DX bio printer, which contained full layers of human skin as previously manufactured by T&R Biofab, South Korea. This organoid was co-cultured with a 5% 3 kDa filtered or non-filtered supernatant of the bacteria in the presence or absence of 20 μg/mL of polyinosinic:polycytidylic acid [poly(I:C)] as stimulus for 48 h, and their supernatants were harvested to measure inflammatory cytokines by ELISA. L-Ascorbic acid (1 mM) was utilized as control.

## Data Availability

The datasets presented in this study can be found in online repositories. The names of the repository/repositories and accession number(s) can be found in the article/**Supplementary Material**.

## Supplementary material

Supplementary Figure 1
**(A)** Raw 264.7 cell line was treated with non-filtered supernatant of the indicated bacteria in the presence or absence of stimuli, total supernatant of pathogenic *C. acnes*, ATCC6919. 24 hours after the treatment, the cell cultured supernatant was harvested and IL-6 inflammatory cytokine was measured via ELISA. **(B)** HaCaT epithelial viability was analyzed 24 hours after the treatment with 5%, 10% or 20% of the *C. acnes* filtrates, and none of the tested samples showed negative effects on cell viability. Student’s unpaired t-test was performed compared to cell only w/stimuli. *<0.05, **<0.01, ***<0.001, ****<0.0001.

Supplementary Figure 2
**(A)** Phylogenetic analysis of 106 *C. acnes* strains. Gene cluster matrix of presence (black)/absence (white) of 4,747 unique genetic contents (columns) included in whole genomes of 106 *C. acnes* strains (rows) is depicted. Missing loci in *C. acnes* type II strains are indicated as red box and unique loci in type II strains are represented by green box in the bottom of gene cluster matrix. Unique loci in type III strains are indicated as blue box in the bottom of gene cluster matrix. Rows represent 106 *C. acnes* genomes colored according to their phylotypes and ribotypes. Columns of gene cluster matrix represent genomic components which were clustered based on their sequence similarity. The presence-absence matrix for the already known four biosynthetic gene clusters (BGCs) and acnecin of *C. acnes* is colored as dark blue-gray. The names of five BGCs are written on the bottom of figure. **(B)** Phylogenetic analysis of 57 type II *C. acnes* strains. Gene cluster matrix of presence (black)/absence (white) of 3,388 unique genetic contents (columns) included in the genomes of 62 *C. acnes* strains (rows) is depicted. Ortholog prevalence in *C. acnes* type II is represented in below the gene cluster matrix. The names of pathogenic strains are colored as red and those of strains obtained from cancer or inflammatory tissues are colored orange. The F0672 is *Cutibacterium modestum* strain. CJIN-1-1, CJIN-1-2 and CJIN-1-3 are type I *C. acnes* strains. The CJIN-3-1 is type III *C. acnes* strain. Rows represent 62 *C. acnes* genomes colored according to their phylotypes and ribotypes. Columns in gene cluster matrix represent genomic components which were clustered based on their similarity.

Supplementary Figure 3
**(A)** Sequencing quality profiles of *C. acnes* transcriptome datasets (eight public and nine internal data) are represented. **(B)** Principal component analysis (PCA) of the trimmed mean of M-value (TMM) normalized CPM values of RNA sequencing data showed that the transcriptomic features of immune regulatory type II strains are distinctly clustered. **(C)** Correlation matrix of gene expression profiles of 17 transcriptome data is depicted. The expressional profiles of CJRS-10655_3 and CJIN-213_3 were excluded from further analysis due to their low correlation coefficients with their related replicates. Heat maps of type II RT2 strains classified as two different groups. Heat maps of the significantly enriched **(D)** and depleted **(E)** expressions in transcriptomic data of immune regulatory strains using Z–scores values of counts were estimated by HTSeq.

Supplementary Figure 4
**(A)** Comparison of genomes among CJRS-10653, ATCC11828 and ATCC6919 strains identified the common genomic variations in the genomic region related to CesSR homologs. **(B)** Multiple alignments of protein sequences of four CesR homologs were performed using MUSCLE. The maximum likelihood tree was built using MEGA X with bootstraps of 1000 replicates. Bootstrap values (as percentages) are denoted at internal nodes. The CesR homologs were aligned with the VraR protein sequences of *S. aureus* and *S. epidermidis*.

Supplementary Figure 5 Metabolomics analysis of two distinct type II RT2 strains. The relative amounts of diverse metabolites related with nucleic acids, vitamins, short chain fatty acids, amino acids and organic acids are shown with standard error. The metabolic compositions obtained from M2 media, culture supernatants of CJIN-2-13, and that of CJRS-10653 are colored as green, red, and blue, respectively.
