# Supplementary figures and images for: Distinct Cutibacterium acnes subspecies defendens strains classified by multi-omics dissection alleviate inflammatory skin lesions of a rosacea-like mouse model
Source: Front Microbiomes. 2024 Oct 21;3:1362408. doi: 10.3389/frmbi.2024.1362408 (PMC12993555; doi:10.3389/frmbi.2024.1362408)

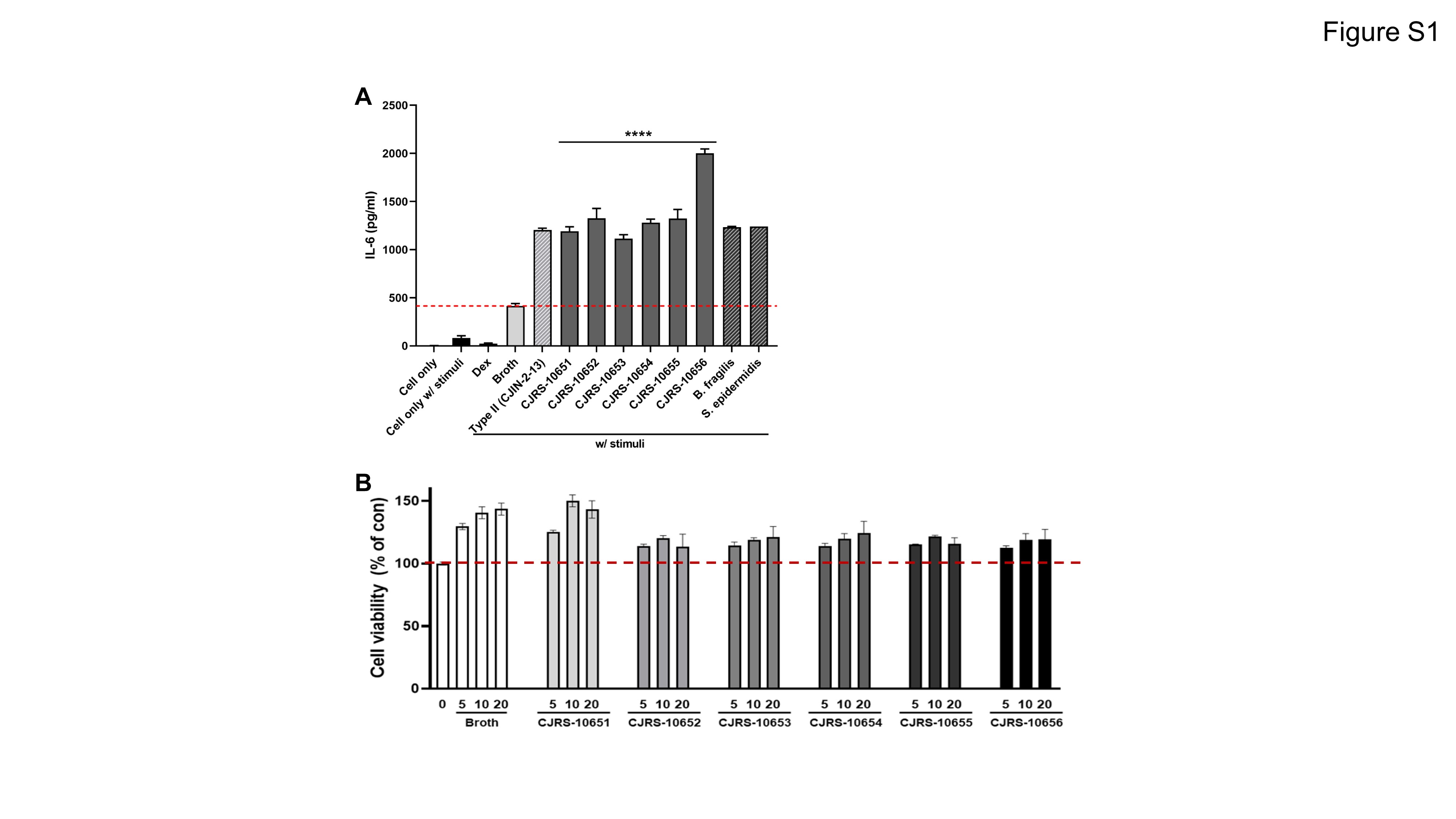

Supplement: Supplementary Figure 1 — (A) Raw 264.7 cell line was treated with non-filtered supernatant of the indicated bacteria in the presence or absence of stimuli, total supernatant of pathogenic C. acnes, ATCC6919. 24 hours after the treatment, the cell cultured supernatant was harvested and IL-6 inflammatory cytokine was measured via ELISA. (B) HaCaT epithelial viability was analyzed 24 hours after the treatment with 5%, 10% or 20% of the C. acnes filtrates, and none of the tested samples showed negative effects on cell viability. Student’s unpaired t-test was performed compared to cell only w/stimuli. *<0.05, **<0.01, ***<0.001, ****<0.0001. [file Image1.jpeg]

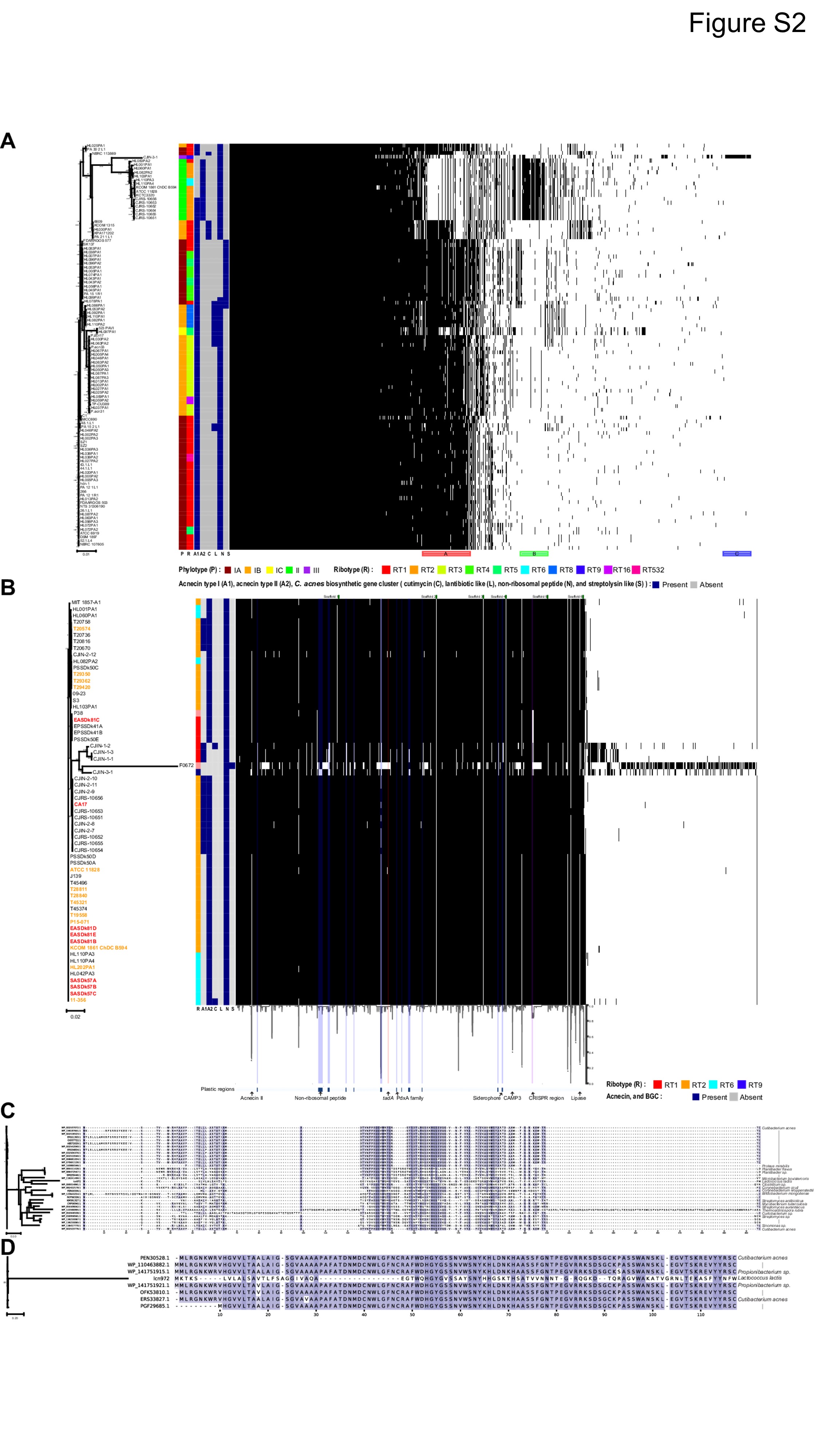

Supplement: Supplementary Figure 2 — (A) Phylogenetic analysis of 106 C. acnes strains. Gene cluster matrix of presence (black)/absence (white) of 4,747 unique genetic contents (columns) included in whole genomes of 106 C. acnes strains (rows) is depicted. Missing loci in C. acnes type II strains are indicated as red box and unique loci in type II strains are represented by green box in the bottom of gene cluster matrix. Unique loci in type III strains are indicated as blue box in the bottom of gene cluster matrix. Rows represent 106 C. acnes genomes colored according to their phylotypes and ribotypes. Columns of gene cluster matrix represent genomic components which were clustered based on their sequence similarity. The presence-absence matrix for the already known four biosynthetic gene clusters (BGCs) and acnecin of C. acnes is colored as dark blue-gray. The names of five BGCs are written on the bottom of figure. (B) Phylogenetic analysis of 57 type II C. acnes strains. Gene cluster matrix of presence (black)/absence (white) of 3,388 unique genetic contents (columns) included in the genomes of 62 C. acnes strains (rows) is depicted. Ortholog prevalence in C. acnes type II is represented in below the gene cluster matrix. The names of pathogenic strains are colored as red and those of strains obtained from cancer or inflammatory tissues are colored orange. The F0672 is Cutibacterium modestum strain. CJIN-1-1, CJIN-1-2 and CJIN-1-3 are type I C. acnes strains. The CJIN-3-1 is type III C. acnes strain. Rows represent 62 C. acnes genomes colored according to their phylotypes and ribotypes. Columns in gene cluster matrix represent genomic components which were clustered based on their similarity. [file Image2.jpeg]

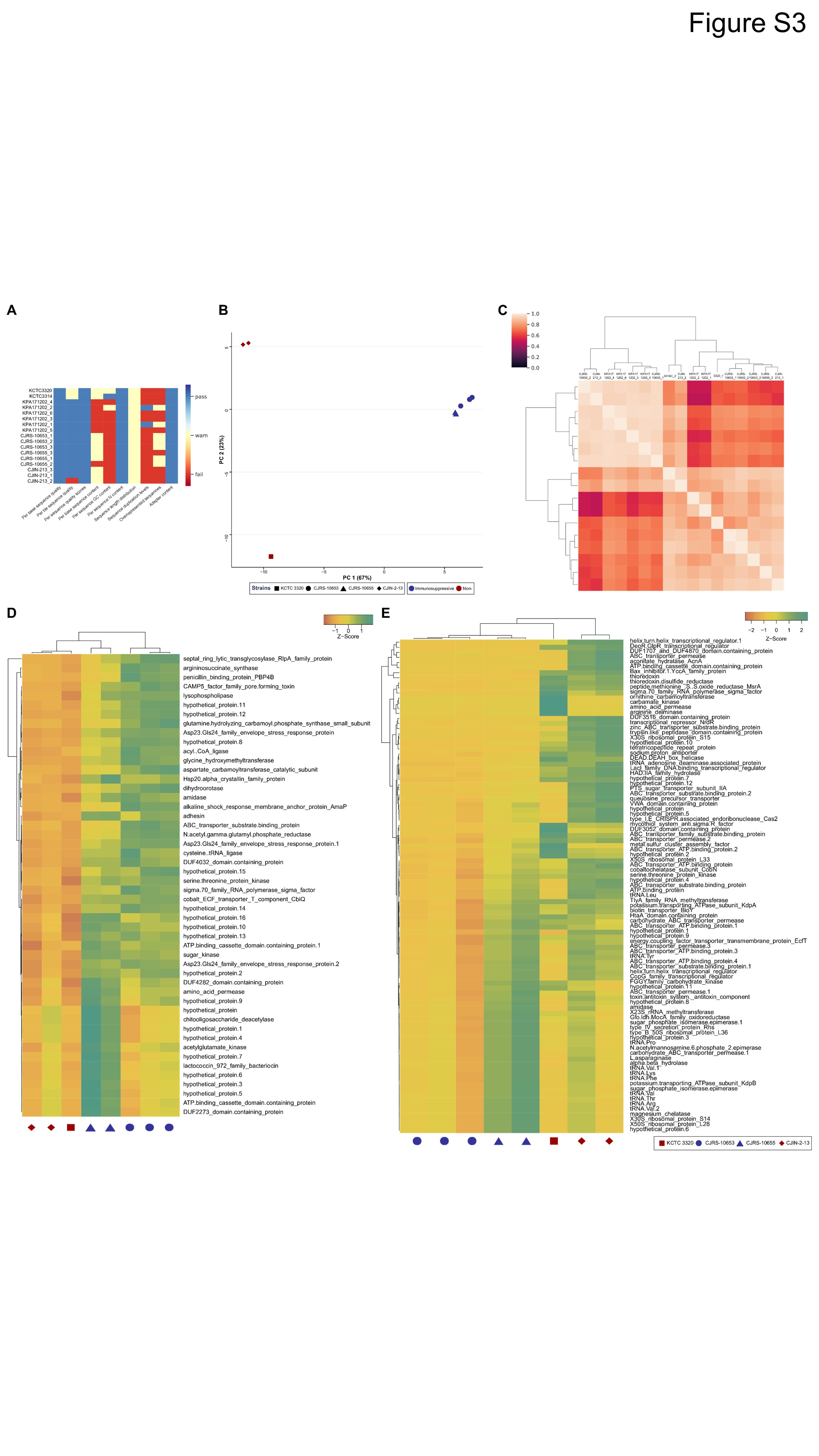

Supplement: Supplementary Figure 3 — (A) Sequencing quality profiles of C. acnes transcriptome datasets (eight public and nine internal data) are represented. (B) Principal component analysis (PCA) of the trimmed mean of M-value (TMM) normalized CPM values of RNA sequencing data showed that the transcriptomic features of immune regulatory type II strains are distinctly clustered. (C) Correlation matrix of gene expression profiles of 17 transcriptome data is depicted. The expressional profiles of CJRS-10655_3 and CJIN-213_3 were excluded from further analysis due to their low correlation coefficients with their related replicates. Heat maps of type II RT2 strains classified as two different groups. Heat maps of the significantly enriched (D) and depleted (E) expressions in transcriptomic data of immune regulatory strains using Z–scores values of counts were estimated by HTSeq. [file Image3.jpeg]

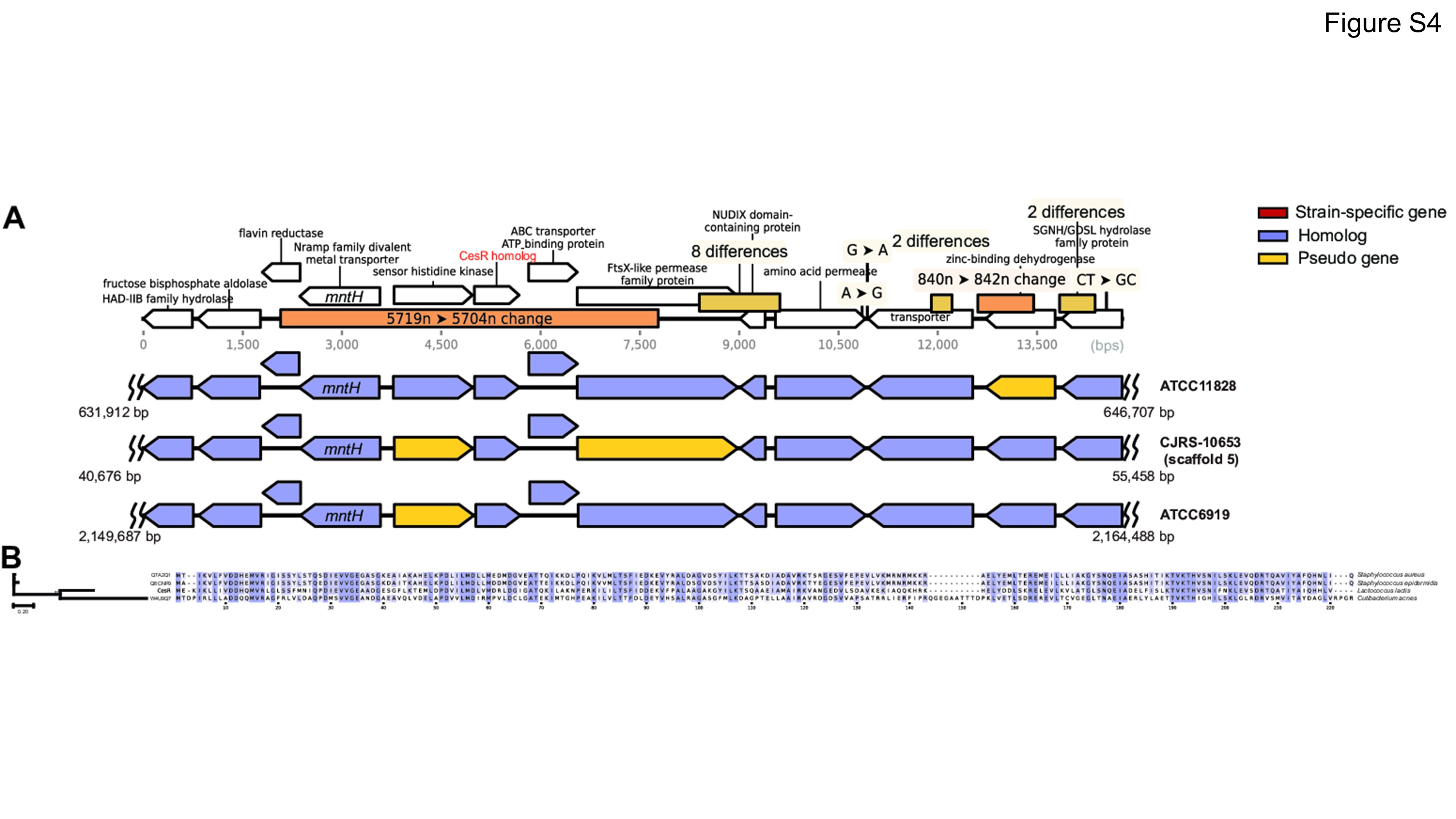

Supplement: Supplementary Figure 4 — (A) Comparison of genomes among CJRS-10653, ATCC11828 and ATCC6919 strains identified the common genomic variations in the genomic region related to CesSR homologs. (B) Multiple alignments of protein sequences of four CesR homologs were performed using MUSCLE. The maximum likelihood tree was built using MEGA X with bootstraps of 1000 replicates. Bootstrap values (as percentages) are denoted at internal nodes. The CesR homologs were aligned with the VraR protein sequences of S. aureus and S. epidermidis. [file Image4.jpeg]

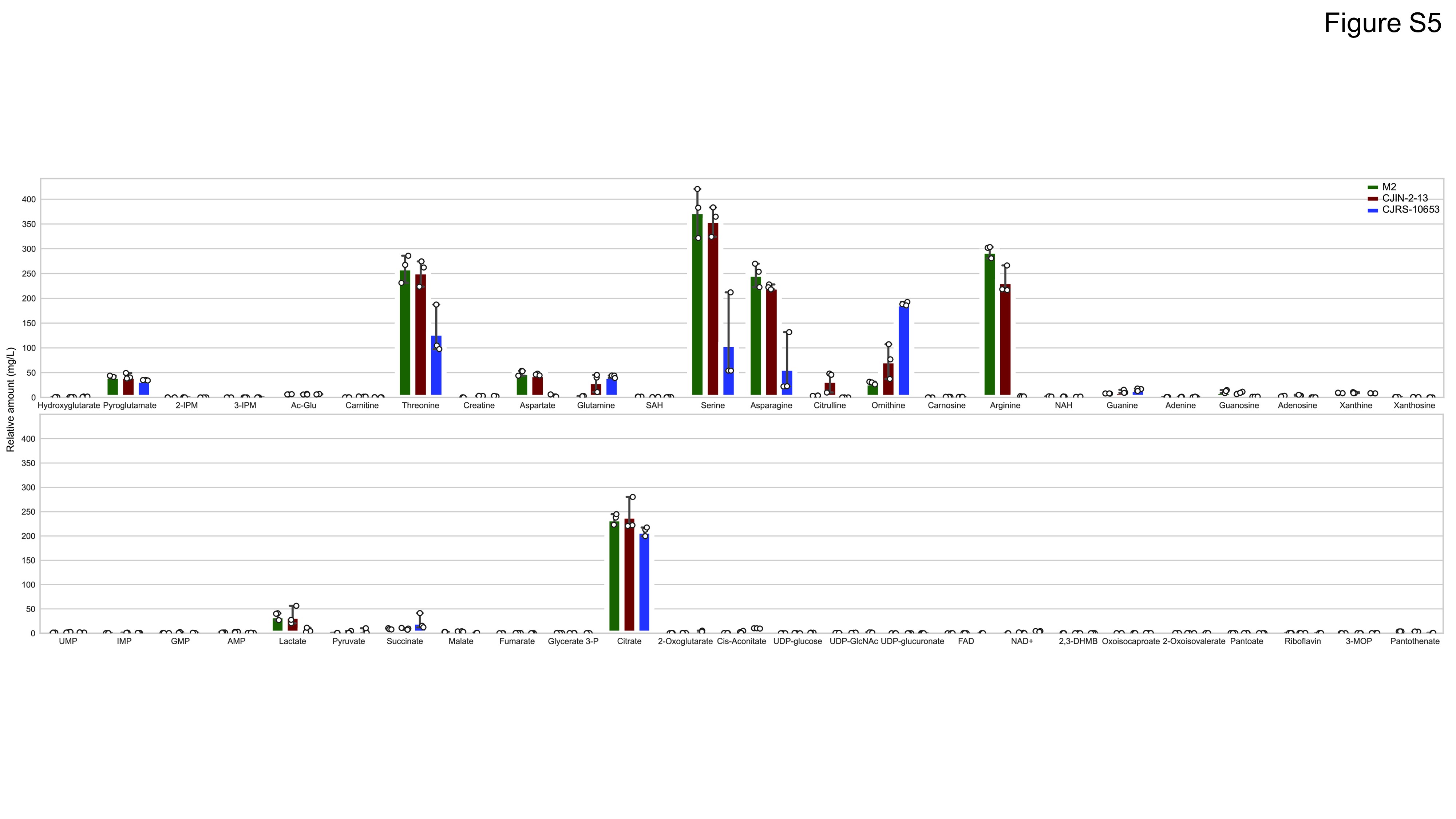

Supplement: Supplementary Figure 5 — Metabolomics analysis of two distinct type II RT2 strains. The relative amounts of diverse metabolites related with nucleic acids, vitamins, short chain fatty acids, amino acids and organic acids are shown with standard error. The metabolic compositions obtained from M2 media, culture supernatants of CJIN-2-13, and that of CJRS-10653 are colored as green, red, and blue, respectively. [file Image5.jpeg]
